# Examining Brain Structures Associated With Emotional Intelligence and the Mediated Effect on Trait Creativity in Young Adults

**DOI:** 10.3389/fpsyg.2018.00925

**Published:** 2018-06-15

**Authors:** Li He, Yu Mao, Jiangzhou Sun, Kaixiang Zhuang, Xingxing Zhu, Jiang Qiu, Xiaoyi Chen

**Affiliations:** ^1^School of Education, Chongqing Normal University, Chongqing, China; ^2^Key Laboratory of Cognition and Personality, Ministry of Education, Southwest University, Chongqing, China; ^3^Faculty of Psychology, Southwest University, Chongqing, China; ^4^Student Mental Health Education and Consultation Center, Chongqing Normal University, Chongqing, China

**Keywords:** emotional intelligence, trait creativity, orbitofrontal cortex, gray matter volume, voxel-based morphometry

## Abstract

Little is known about the association between emotional intelligence (EI) and trait creativity (TC), and the brain structural bases which involves. This study investigated the neuroanatomical basis of the association between EI and TC which measured by the Schutte self-report EI scale and the Williams creativity aptitude test. First, the voxel-based morphometry (VBM) analysis was used to explore the brain structures which is closely related to EI in a large young sample (*n* = 213). The results showed that EI was positively correlated with the regional gray matter volume (rGMV) in the right orbitofrontal cortex (OFC), which is regarded as a key region of emotional processing. More importantly, further mediation analysis revealed that rGMV in the right OFC partially mediated the association between EI and TC, which showed the OFC volume could account for the relationship between EI and TC. These findings confirmed the close relationship between EI and TC, and highlighted that the brain volumetric variation in the OFC associated with the top-down processing of emotion regulation, which may play a critical role in the promotion of TC. Together, these findings contributed to sharpening the understanding of the complex relationship between EI and TC from the perspective of brain structural basis.

## Introduction

Emotional intelligence (EI) refers to the ability to reason and analyze emotions accurately, as well as utilize emotions and emotional knowledge to enhance thought and action (Mayer et al., 2008), which also reflects individual’s ability to perceive, regulate, and utilize emotion (Salovey and Mayer, 1990). Individuals with high EI are more likely to gain more beneficial outcomes (Parke et al., 2015), such as closer relationship (Brackett et al., 2006), better social problem solving (Barbey et al., 2014), higher well-being and life satisfaction (Kong and Zhao, 2013), better work performance and higher academic achievement (Rivers et al., 2012; Libbrecht et al., 2014). On the contrary, the deficiency of EI always pose a threat to mental and body health, which may cause psychological distress (Hertel et al., 2009), gaming and Internet abuse (Parker et al., 2013), anxiety and depression (Salguero et al., 2012; Zavala and Lopez, 2012).

Emerging neuroscience studies have revealed that several emotion-related regions may correlate with EI, such as the anterior insula (AI), amygdala, orbitofrontal cortex (OFC), anterior cingulate cortex (ACC), and ventromedial prefrontal cortex (vmPFC) (Frith and Frith, 2007; Blakemore, 2008; Krueger et al., 2009; Kreifelts et al., 2010; Koven et al., 2011; Takeuchi et al., 2011, 2013a,b; Pan et al., 2014; Tan et al., 2014; Hogeveen et al., 2016b). The AI and ACC have been regarded to play a critical in the generating of emotional awareness, an important ability to perceive feelings of oneself (Medford and Critchley, 2010; Hogeveen et al., 2016a). For the ability to understand other’s emotions, some evidence showed that the amygdala and the vmPFC may aim to ensure the recognition of facial expressions (Vuilleumier et al., 2004; Wolf et al., 2014). The OFC has been regarded as a core region of emotional assessment and emotional regulation, especially which adjusts emotional expression by reasonably evaluating the emotional salient stimuli and regulating the subjective emotional experience (Kringelbach, 2005; Roelofs et al., 2009). Prior studies also found the OFC is associated with inhibitory control, which may help to regulate negative emotion effectively, reduce maladaptive behavior, and enhance behavior flexibility (Roberts and Wallis, 2000; Rudebeck et al., 2013). In addition, studies on human brain lesion further confirmed several brain regions related to individual’s emotional ability. Patients with substantial lesion in the AI exhibited significantly higher levels of alexithymia (Hogeveen et al., 2016a). Alexithymia can be characterized by dysfunction in emotional awareness, social interaction, and interpersonal relationship (Bagby et al., 1994), which was negatively correlated with EI (Baughman et al., 2013; Onur et al., 2013). Other research showed that patients with damages in the OFC exhibit lower EI score and behaved handicaps in emotional regulation compared with the healthy group, which significantly impaired subjective emotional experience and adaptive behavior (Bar-On et al., 2003; Beer et al., 2003; Hornak et al., 2003; Krueger et al., 2009; Hogeveen et al., 2016b). At the extremes, the damage or dysfunction in the OFC may result in ‘acquired sociopathic’ behavior (Saver and Damasio, 1991).

Combining the above studies, it is obvious that EI plays an important role in emotional processing, interpersonal communication, academic achievement, work performance and so on, which reveals the ability to perceive, regulate, and utilize the emotions of oneself and others is vital for daily life. As an extremely important human activity, creative behavior would be affected by emotion as well (Higgins et al., 1992; Fong, 2006; Hoffmann and Russ, 2012; Kim et al., 2013; Ding et al., 2014; Hao et al., 2017), while previous studies also showed a significant positive correlation between EI and creativity (Guastello et al., 2004; Barczak et al., 2010; Carmeli et al., 2014; Parke et al., 2015; Sahin et al., 2016; Toyama and Mauno, 2017). For example, Barczak et al. (2010) investigated the influence of team EI on team creativity in a young sample. They found team EI enhanced team creativity by promoting team trust and developing a collaborative culture of the team. Researchers also investigated the impact of EI on creativity in workplace, Carmeli et al. (2014) found that employees with higher EI tend to exhibit a higher level of generosity, which fosters a sense of vigor, and further results in the enhancement of creativity. In addition, based on the affective information processing theory, a recent study showed that the emotional regulation of EI allows employees to keep more positive affect when facing a complex problem situation, and the emotional facilitation of EI enables employees to utilize positive affect to promote creativity (Parke et al., 2015). Guastello et al. (2004) investigated the relationships among EI, mood disorders, and creativity, results showed that EI and creativity were higher among people with mood disorders who completed treatment relative to people in treatment, which means EI may improve creativity by offsetting emotional disorders and maintaining positive affect (Guastello et al., 2004). The above findings revealed a positive effect of EI on creativity.

As we know, numerous outstanding creators like Albert Einstein always possess great creative potential that makes them unique and acquires more creative achievements. Creative potential can be regarded as a multidimensional composite consists of some aspects related to cognition and others related to personality (Gough, 1979; Piffer, 2012; Li et al., 2015; Silvia et al., 2016). Creative cognition refers to the cognitive processes that occur in the generation and evaluation of creative ideas and products, such as divergent thinking, whereas creative traits might be a series of aptitude or personality variables (e.g., curiosity, openness to experience, and imagination) that also could integrate other factors such as psychopathological traits and genetic impacts (Zeng et al., 2009; Piffer, 2012; Li et al., 2015; Zhuang et al., 2017). In line with precious studies (Li et al., 2015; Zhuang et al., 2017), we focused on personality or aptitude aspects of the creative trait that usually assessed using the Williams creativity aptitude test (WCAT). These aptitudes (trait creativity, TC) have a positive impact on creative thinking and creative problem solving (Sternberg, 1999). Prior study also suggested that TC acts as a valid predictor of creative achievement in real life (Feist and Barron, 2003). Most existing studies, however, have only focused on the association between EI and creative cognition, ignoring the association between EI and TC, as well as the brain structure bases which involves. Thus, the present study aimed to investigate the association between EI and TC, and elucidates the brain neural substrates between them in a large young sample.

A recent voxel-based morphometry (VBM) study revealed that TC (as measured by WCAT) was associated with emotion-related brain structures, such as the regional gray matter volume (rGMV) of the OFC, hippocampus, and amygdala (Zhuang et al., 2017), while these regions are closely related to emotional processing. Intriguingly, another brain structure study also found that EI displayed a close correlation with the rGMV in the insula, OFC, and the parahippocampal gyrus (Tan et al., 2014). These similar brain regions involved in EI and TC suggest that they may share a common brain structure basis. Moreover, individuals with higher EI who exhibit more excellent ability of emotional processing and tend to be critical thinkers (Yao et al., 2017), which may help to creative problem solving (Eggers et al., 2017). Taken together, the above findings may reveal a close relationship between EI and TC, and the underlying similar brain structures that they both involved. However, to our knowledge, there is no direct evidence have clarified the complex association between them. In this study, we examined the association between EI and TC, as measured by the Schutte Self-Report Emotional Intelligence Scale and WCAT, respectively. Then, the VBM analysis was used to identify the rGMV related to EI at the whole-brain level. Considering EI refers to a set of emotional abilities (e.g., emotional regulation, utilization of emotions, appraisal of emotions and so on), we hypothesized that EI would be closely associated with the rGMV in emotion-related areas such as the OFC, ACC, and the amygdala. Furthermore, we conducted a mediation analysis to explore whether brain structures could account for the association between EI and TC, because EI and TC may share similar brain structure bases (Tan et al., 2014; Zhuang et al., 2017).

## Materials and Methods

### Participants

A total of 225 right-handed, healthy subjects from the Southwest University in China participated in the study as part of our ongoing project to examine the associations between brain, creativity, and mental health. Seven subjects were excluded because of incomplete behavior data, and five participants were excluded due to extraordinary motion artifacts. Therefore, 213 participants were included in analyses (103 males and 110 females; mean age = 20.0 ± 1.3; ranged from 17 to 27). Based on a self-report questionnaire survey before the scan, none of them had a history of psychiatric or neurological illness, or substance abuse. This study was granted by the Institutional Review Board of Southwest University Imaging Center for Brain Research, and all participants signed the written informed consents.

### Emotional Intelligence Scale

The Schutte Self-Report Emotional Intelligence Scale (SSREIS) is an effective self-report EI assessment that developed by Schutte et al. (1998). The SSREIS in Chinese version has been widely used in Chinese populations and includes four dimensions: regulation of emotions, utilization of emotions, self-emotion appraisal, and others’ emotion appraisal (Wang, 2002). Each item was used a five-point scale ranging from “not true of me” to “very often true of me.” The total score is calculated by adding the answers of all the items, which represents the level of EI. The Cronbach’s α in the present study was 0.79.

### Williams Creativity Aptitude Test

Trait creativity was measured by the WCAT, which is part of the creativity assessment packet (Williams, 1980). The Chinese version developed by Lin and Wang (1994), which consists of 50 items and contains four domains: challenge, imagination, curiosity, and risk-taking. Participants were instructed to rate the extent to which they agree or disagree with each item using a six-point Likert scale. The total score was calculated by adding the answers of all the items. The higher score individuals have, the greater aptitude for creativity they exhibit. This scale showed a good reliability and validity in prior studies (Lin and Wang, 1994; Hwang et al., 2007; Li et al., 2015). The Cronbach’s α in the present study was 0.79.

### General Intelligence

In order to dispel the impact of general intelligence on EI and creativity, the Chinese version of the Combined Raven’s Test-Rural (CRT-RC3) was used to measure individuals’ general intelligence. The CRT-RC3 contains the Raven’s colored progressive matrix (A, B, and AB sets) and Raven’s standard progressive matrix (C, D, and E sets), which consist of 72 non-verbal items and each item requires participants to select the best answer from six or eight alternatives to complete the missing matrix. This scale has been widely used in Chinese populations and previous researches have reported that this test exhibits a good degree of reliability and validity in measuring general intelligence (Wang, 2007).

### Magnetic Resonance Image Data Acquisition

Imaging data were collected by using a 12-channel head coil on a Siemens 3 T Trio scanner (Siemens Medical Systems, Erlangen, Germany). High-resolution T1-weighted structural images were acquired with a magnetization-prepared rapid gradient echo (MPRAGE) sequence: TR = 1900 ms; TE = 2.52 ms; flip angle = 9°; FOV = 256 mm × 256 mm; slices = 176; thickness = 1.0 mm; voxel size = 1 mm × 1 mm × 1 mm.

### Voxel-Based Morphometry Analysis

The MR images were processed with the VBM toolbox using SPM8^1^ implemented in MATLAB R2010a (Math Works Inc., Natick, MA, United States). Firstly, all images from each subject were displayed in SPM8 to screen for artifacts or gross anatomical abnormalities. In the process of registration, the MR images were manually reoriented to the anterior commissure to enhance registration. Then, the MR images were segmented into gray matter, white matter, and cerebrospinal fluid by using the new segmentation toolbox in SPM8. Subsequently, we performed DARTEL for registration, normalization, and modulation (Ashburner, 2007). To ensure that regional differences in the total amount of gray matter were conserved, the image intensity of each voxel was modulated by Jacobian determinants derived from spatial normalization (Good et al., 2001). Additionally, the registered images were transformed to MNI space. Finally, in order to improve the signal-to-noise ratio, an 8-mm full-width at half-maximum (FWHM) Gaussian kernel was used to smooth the modulated images.

### Statistical Analysis

Voxel-based morphometry analysis was performed in SPM8. We used multiple linear regression analysis at the whole-brain level to determine whether the rGMV was associated with individual differences in EI score. In order to eliminate the potential effects of possible confound variables, gender, age, general intelligence, and total GMV were controlled as no interest variables. An absolute threshold masking of 0.2 was used to minimize the boundary effects between gray matter and white matter, which means signal intensity values of voxels with gray matter lower than 0.2 were removed from the analysis. The voxel-level family-wise error (FWE) method was used at the whole-brain level, and the significance threshold was set at *p* < 0.05, corrected for multiple comparisons.

### Mediation Analysis

To examine whether the specific rGMV could explain the association between EI and TC, mediation analysis was performed by using SPSS macro with 0.95 confidence level and 5000 bootstrap sample (Preacher and Hayes, 2004, 2008). The total effect of EI on TC (path c) consists of two parts, namely, the direct effect of EI on TC after controlling for the rGMV of the right OFC (path c’) and the indirect effect of EI on TC through the OFC volume (path a × b). The mediation analysis aims to evaluate whether the indirect effect is significant. If the confidence interval (CI) does not include zero, which means the rGMV of the OFC mediated the association between EI and TC. In addition, gender, age, general intelligence, and total GMV were controlled as covariates in the model.

## Results

### Behavioral Results

The average, standard deviation, and Person correlation of all variables for this sample are presented in **Table 1**. The statistical software SPSS 22.0 was used to analyze all behavioral data, and the Pearson correlation coefficient of EI and TC was calculated by controlling for gender, age, and general intelligence. As indicated in **Table 1**, there were weakly but significantly positive correlations between the subscale scores of the SSREIS and the subscale scores of the WCAT. In addition, the results also showed that the total score of the SSREIS was strongly and significantly positively associated with the total score of the WCAT (*r* = 0.42, *p* < 0.001), which revealed a close relationship between EI and TC.

**Table 1:** The means, standard deviations, and correlations of scores on SSREIS and WCAT (*n* = 213).

	Mean (*SD*)	Range	Gender	Age	Raven	ME	UE	SA	AEO	TEI	Risk-taking	Curiosity	Imagination	Challenge	TTC
Gender	1.52 (0.50)	1–2	–	-0.20**	0.04	0.01	-0.08	0.10	-0.05	-0.01	-0.09	-0.19**	-0.05	-0.13	-0.16*
Age	19.95 (1.28)	17–27		-	0.01	-0.06	-0.17*	-0.14*	-0.06	-0.15*	-0.17*	0.01	-0.03	-0.26**	-0.14*
Raven	66.25 (3.28)	50–72			-	0.08	-0.02	0.06	0.04	0.05	0.04	0.11	-0.02	-0.06	0.03
**SSREIS**															
ME	17.86 (2.32)	12–23				-	0.37***	0.40***	0.46***	0.79***	0.30***	0.29***	0.27***	0.24***	0.38***
UE	19.33 (2.24)	13–25					-	0.22**	0.33***	0.68***	0.19**	0.16*	0.20**	0.16*	0.24***
SA	20.69 (1.99)	16–25						-	0.31***	0.66***	0.25***	0.28***	0.10	0.21**	0.29***
AEO	14.85 (2.03)	10–20							-	0.72***	0.26***	0.24***	0.16*	0.25***	0.31***
TEI	72.73 (6.17)	59–92								-	0.35***	0.34***	0.26***	0.30***	0.42***
**WCAT**															
Risk-taking	24.49 (2.69)	18–31									-	0.34***	0.45***	0.51***	0.75***
Curiosity	32.27 (3.68)	23–41										-	0.28***	0.42***	0.72***
Imagination	26.57 (3.49)	18–35											-	0.36***	0.73***
Challenge	28.37 (2.86)	21–35												-	0.74***
TTC	111.69 (9.35)	89–134													-

Gender: male = 1, female = 2; SD, standard deviation; ME, Monitor of Emotions; UE, Utilization of Emotions; SA, Social Ability; AEO, Appraisal of Emotions in Others; TEI, total score of emotional intelligence; TTC, total score of trait creativity; SSREIS, Schutte Self-Report Emotional Intelligence Scale; WCAT, Williams Creativity Aptitude Test. ^∗^*p* < 0.05. ^∗∗^*p* < 0.01. ^∗∗∗^*p* < 0.001.

### VBM Results

**FIGURE 1:**
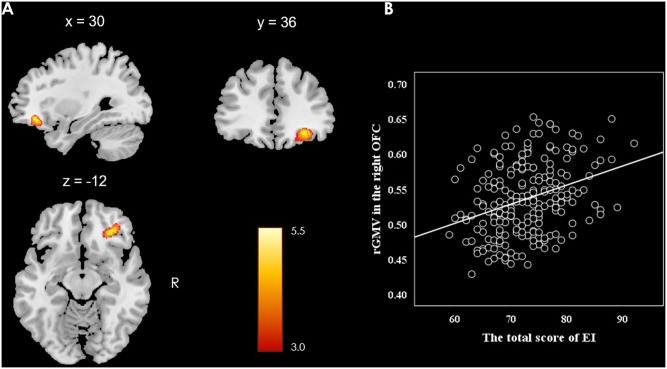
Brain regions of positive correlation between GMV and EI. **(A)** The right OFC exhibited significant positive correlation with total score of EI as measured by the SSREIS (shown at *t* > 3 for visualization purposes), gender, age, general intelligence and total GMV were regressed out. **(B)** Scatter plots showed the correlation between EI and rGMV in the right OFC (*r* = 0.33, *p* < 0.001).

This study investigated the relationship between the rGMV and individual difference in EI (measured by SSREIS) at the whole-brain level. In order to eliminate the potential effects of possible confound variables, gender, age, general intelligence, and total GMV were controlled as no interest covariates and regressed out. At the whole-brain level, multiple regression analysis revealed a positive relationship between total score of the SSREIS and the rGMV in the right OFC (Brodmann area: 11; MNI coordinate: 30, 36, -12; cluster size = 95; *r* = 0.33; *t* = 5.40; *p* = 0.001, FWE corrected; see **Figure 1**). There was no significant negative correlation between total score of the SSREIS and any other brain regions (*p* > 0.05, FWE corrected), subscale scores of the SSREIS also showed an insignificant correlation with any other brain regions as well (*p* > 0.05, FWE corrected).

After identifying potential neural correlations of EI, we used the right OFC that is significantly correlated with EI from the VBM analysis to further examined the relationship between WCAT and OFC, the mean gray matter volume extracted by using the REX toolbox developed by the LKM experts^2^. Correlation analysis showed that rGMV in the OFC was positively correlated with imagination (*r* = 0.13; *p* = 0.05), curiosity (*r* = 0.24; *p* < 0.001), challenge (*r* = 0.28; *p* < 0.01), risk-taking (*r* = 0.30; *p* < 0.001), and TC total score (*r* = 0.32; *p* < 0.001) as measured by WCAT. After controlling gender, age, and Raven’s score, the correlation results stood robust (imagination, *r* = 0.12, *p* > 0.05; curiosity, *r* = 0.20, *p* = 0.005; challenge, *r* = 0.22, *p* = 0.001; risk-taking, *r* = 0.26, *p* < 0.001; TC total score, *r* = 0.26, *p* < 0.001).

### Mediation Results

Based on behavior and VBM results, we performed a mediation analysis to further explore whether EI affects TC through rGMV in the right OFC. In the present study, we chose the EI total score measured by SSREIS as independent variable, TC (imagination, curiosity, challenge, risk-taking, and total score) measured by WCAT as dependent variables, and rGMV in the right OFC as the mediator. In addition, we also regarded gender, age, general intelligence, and total GMV as covariates for all mediation analyses.

**FIGURE 2:**
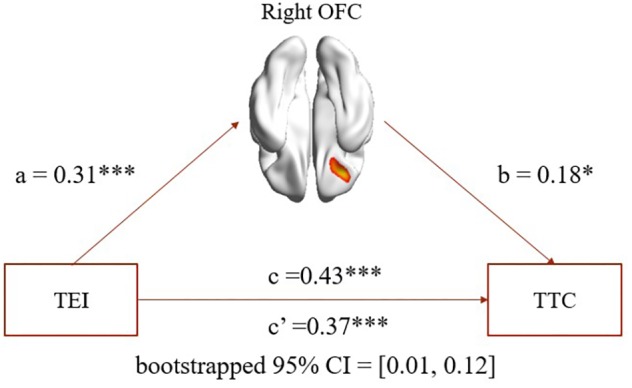
Mediation analysis. Path a, path b, and path c are significant, and path c’ is significantly smaller than path c (bootstrapped 95% CI: 0.01–0.12). The mediation results suggest that rGMV in the right OFC partially mediated the association between TEI and TTC. TEI, total score of emotional intelligence; TTC, total score of trait creativity. ^∗^*p* < 0.05. ^∗∗^*p* < 0.01. ^∗∗∗^*p* < 0.001

As expected, mediation analysis revealed that (1) rGMV in the right OFC partially mediated the association between EI total score and TC total score (path a = 0.31, *p* < 0.001; path b = 0.18, *p* = 0.02; path a × b = 0.06, bootstrapped 95% CI = 0.01, 0.12; see **Figure 2**); (2) rGMV in the right OFC partially mediated the association between EI total score and curiosity (path a = 0.31, *p* < 0.001; path b = 0.16, *p* = 0.04; path a × b = 0.05, bootstrapped 95% CI = 0.002, 0.114); (3) rGMV in the right OFC partially mediated the association between EI total score and risk-taking (path a = 0.31, *p* < 0.001; path b = 0.16, *p* = 0.04; path a × b = 0.05, bootstrapped 95% CI = 0.006, 0.115).

## Discussion

The present study investigated the neural basis of EI and the relationship among EI, brain structure, and individual TC as measured by WCAT in a large young sample. Behavioral results showed that EI was positively correlated with TC, and it’s noteworthy that there was a strong and significant positive association between total score of the SSREIS and the total score of the WCAT. In addition, VBM results revealed that rGMV in the right OFC displayed a positive correlation with EI. More importantly, further mediation analysis showed that the right OFC volume partially mediated the association between EI and TC.

Behavioral results confirmed that different dimensions of EI was positively correlated with TC in varying degrees, especially there was a strong and significant positive relationship between the overall level of EI and TC, which is consistent with previous findings (Guastello et al., 2004). Several investigations have showed that the higher level of EI individuals have, the better ability to perceive, regulate, and utilize the emotions of oneself and others they exhibit (Brackett et al., 2006), which always lead to maintain a good mood in daily life (Hertel et al., 2009). Meanwhile, positive affect is also linked with the improvement of TC (Guastello et al., 2004), that is, individual’s creativity is generally activated in a positive emotional state, which contributes to producing more creative behaviors and higher creative achievements (Ding et al., 2014; Libbrecht et al., 2014). Based on these findings, it is reasonably speculated that individuals with greater levels of EI tend to exhibit higher TC.

Voxel-based morphometry results exhibited that EI was significantly positively correlated with brain regions mainly located in the right OFC, which has been considered to play a crucial role in emotional processing, sensory integration, and reward-driven system (Kringelbach, 2005). Stalnaker et al. (2015) also suggested that the OFC is critical for signaling emotions as well. In addition, studies on human brain lesion provided evidence for the confirmation of emotional function related to the OFC. For example, patients with lesion in the OFC performed handicaps in identifying emotional face and voice (Hornak et al., 2003), as well as effective emotional regulation (Beer et al., 2003; Hogeveen et al., 2016b), which significantly impaired interpersonal communication and social behavior (Hornak et al., 2003). These findings contribute to the deeper understanding of the OFC guides adaptive behavior and emotional experience (Stalnaker et al., 2015). Furthermore, previous VBM studies have demonstrated that individuals with generalized anxiety disorder have significantly less rGMV in the OFC (Moon and Jeong, 2015), while higher EI can predict less anxiety and depression (Salguero et al., 2012; Zavala and Lopez, 2012), which revealed that the OFC volume may be closely related to EI, particularly the emotional regulation of EI (Tan et al., 2014). Spontaneous brain activity during resting state also found a close relationship between EI and OFC (Pan et al., 2014). Combined with these findings, individuals with higher EI generally exhibit better emotional processing ability, especially in the top–down aspects of emotional regulation, which is closely related to the volumetric variation in the OFC.

Interestingly, mediation results revealed that rGMV in the OFC partially mediated the association between EI and TC. Based on previous studies, individuals with high EI generally exhibited excellent emotional regulation ability and benign social cognitive functioning (Brackett et al., 2006; Hertel et al., 2009), that may play a major role in promoting the tendency to engage in creativity (Guastello et al., 2004; Ding et al., 2014; Parke et al., 2015). From the perspective of the brain structure variations, the OFC is closely related to emotional processing (Kringelbach, 2005), especially in the top-down aspects of emotional regulation (Petrovic et al., 2016; Silvers et al., 2016). Emotional regulation is essential for the maintenance of positive affect that further contribute to promoting creativity (Parke et al., 2015), our results also showed the OFC volume associated with TC, which was in line with a recent VBM study. Zhuang et al. (2017) found that TC was positively correlated with emotion-related brain region, especially in the OFC (Zhuang et al., 2017); another neuroimaging study also reported that the higher levels of self-report creativity individuals have, the larger cortical surface of the OFC they exhibit (Bashwiner et al., 2016). These findings provided evidence for the close association between the OFC and TC, and further showed the OFC volume could account for the relationship between EI and TC. Taken together, the above discussions suggested that the larger volume in the OFC associated with EI reflects enhancement of emotional processing ability, especially in the top–down aspects of emotional regulation, which contributes to the promotion of TC.

Several limitations of this study should be mentioned. First, although the sample size is relatively large compared with other studies (Koven et al., 2011; Killgore et al., 2012), all participants of the present study consisted of healthy, young undergraduates, which may restrain the generalizability of these findings. In addition, behavior data mainly relied on self-report questionnaires, in spite of previous researches have shown that these questionnaires used in our study have high reliability and validity, the results may also be vulnerable to social desirability. The use of multiple methods and repeated measurements may reduce the impact of subjectivity. Equally important, the cross-sectional design limits causal inference, and the implementation of longitudinal studies may be needed to further clarify causal directions.

## Conclusion

The present study found that the gray matter volumetric variation in the right OFC positively correlated with EI. Moreover, further mediation analysis revealed that the OFC volume partially mediated the association between EI and TC, suggested that the larger volume in the OFC associated with EI reflects enhancement of emotional processing ability, especially in the top-down aspects of emotional regulation, which contributes to the promotion of TC. These results help sharpen the understanding of the relationship between EI and TC from the perspective of neural substrates.

## Author Contributions

LH, JQ, and XC designed and conducted the study. LH, YM, JS, KZ, and XZ analyzed the data. LH, YM, and XC drafted the manuscript. JQ and XC provided critical revisions.

## Conflict of Interest Statement

The authors declare that the research was conducted in the absence of any commercial or financial relationships that could be construed as a potential conflict of interest.

## Footnotes

1 www.fil.ion.ucl.ac.uk/spm

2 http://web.mit.edu/swg/software.htm

## References

[B1] Ashburner J. (2007). A fast diffeomorphic image registration algorithm. *Neuroimage* 38 95–113. 10.1016/j.neuroimage.2007.07.007 17761438

[B2] Bagby R. M. Parker J. D. A. Taylor G. J. (1994). The 20-item toronto alexithymia scale:1. Item selection and cross-validation of the factor structure. *J. Psychosom. Res.* 38 23–32. 10.1016/0022-3999(94)90005-1 8126686

[B3] Barbey A. K. Colom R. Paul E. J. Chau A. Solomon J. Grafman J. H. (2014). Lesion mapping of social problem solving. *Brain* 137 2823–2833. 10.1093/brain/awu207 25070511 PMC4163035

[B4] Barczak G. Lassk F. Mulki J. (2010). Antecedents of team creativity: an examination of team emotional intelligence, team trust and collaborative culture. *Creat. Innov. Manage.* 19 332–345. 10.1111/j.1467-8691.2010.00574.x

[B5] Bar-On R. Tranel D. Denburg N. L. Bechara A. (2003). Exploring the neurological substrate of emotional and social intelligence. *Brain* 126 1790–1800. 10.1093/brain/awg177 12805102

[B6] Bashwiner D. M. Wertz C. J. Flores R. A. Jung R. E. (2016). Musical creativity “revealed” in brain structure: interplay between motor, default mode, and limbic networks. *Sci. Rep.* 6:20482. 10.1038/srep20482 26888383 PMC4757893

[B7] Baughman H. M. Schermer J. A. Veselka L. Harris J. Vernon P. A. (2013). A behavior genetic analysis of trait emotional intelligence and alexithymia: a replication. *Twin Res. Hum. Genet.* 16 554–559. 10.1017/thg.2012.151 23298794

[B8] Beer J. S. Heerey E. A. Keltner D. Scabini D. Knight R. T. (2003). The regulatory function of self-conscious emotion: insights from patients with orbitofrontal damage. *J. Pers. Soc. Psychol.* 85 594–604. 10.1037/0022-3514.85.4.594 14561114

[B9] Blakemore S. J. (2008). The social brain in adolescence. *Nat. Rev. Neurosci.* 9 267–277. 10.1038/nrn2353 18354399

[B10] Brackett M. A. Rivers S. E. Shiffman S. Lerner N. Salovey P. (2006). Relating emotional abilities to social functioning: a comparison of self-report and performance measures of emotional intelligence. *J. Pers. Soc. Psychol.* 91 780–795. 10.1037/0022-3514.91.4.780 17014299

[B11] Carmeli A. McKay A. S. Kaufman J. C. (2014). Emotional intelligence and creativity: the mediating role of generosity and vigor. *J. Creat. Behav.* 48 290–309. 10.1002/jocb.53

[B12] Ding X. Q. Tang Y. Y. Tang R. X. Posner M. I. (2014). Improving creativity performance by short-term meditation. *Behav. Brain Funct.* 10:9. 10.1186/1744-9081-10-9 24645871 PMC3994657

[B13] Eggers F. Lovelace K. J. Kraft F. (2017). Fostering creativity through critical thinking: the case of business start-up simulations. *Creat. Innov. Manage.* 26 266–276. 10.1111/caim.12225

[B14] Feist G. J. Barron F. X. (2003). Predicting creativity from early to late adulthood: intellect, potential, and personality. *J. Res. Pers.* 37 62–88. 10.1016/s0092-6566(02)00536-6

[B15] Fong C. T. (2006). The effects of emotional ambivalence on creativity. *Acad. Manag. J.* 49 1016–1030. 10.1016/j.jad.2014.10.040 25577160

[B16] Frith C. D. Frith U. (2007). Social cognition in humans. *Curr. Biol.* 17 724–732. 10.1016/j.cub.2007.05.068 17714666

[B17] Good C. Johnsrude I. Ashburner J. Henson R. Friston K. Frackowiak R. (2001). A voxel-based morphometric study of ageing in 465 normal adult human brains. *Neuroimage* 1 21–36. 10.1006/nimg.2001.0786 11525331

[B18] Gough H. G. (1979). Creative personality scale for the adjective check list. *J. Pers. Soc. Psychol.* 37 1398–1405. 10.1037/0022-3514.37.8.1398

[B19] Guastello S. J. Guastello D. D. Hanson C. A. (2004). Creativity, mood disorders, and emotional intelligence. *J. Creat. Behav.* 38 260–281. 10.1002/j.2162-6057.2004.tb01244.x

[B20] Hao N. Xue H. Yuan H. Wang Q. Runco M. A. (2017). Enhancing creativity: proper body posture meets proper emotion. *Acta Psychol.* 173 32–40. 10.1016/j.actpsy.2016.12.005 27992759

[B21] Hertel J. Schutz A. Lammers C. H. (2009). Emotional intelligence and mental disorder. *J. Clin. Psychol.* 65 942–954. 10.1002/jclp.20597 19437504

[B22] Higgins L. F. Qualls S. H. Couger J. D. (1992). The role of emotions in employee creativity. *J. Creat. Behav.* 26 119–129. 10.1002/j.2162-6057.1992.tb01167.x

[B23] Hoffmann J. Russ S. (2012). Pretend play, creativity, and emotion regulation in children. *Psychol. Aesthet. Creat. Arts* 6 175–184. 10.1037/a0026299

[B24] Hogeveen J. Bird G. Chau A. Krueger E. Grafman J. (2016a). Acquired alexithymia following damage to the anterior insula. *Neuropsychologia* 82 142–148. 10.1016/j.neuropsychologia.2016.01.021 26801227 PMC4752907

[B25] Hogeveen J. Salvi C. Grafman J. (2016b). ‘Emotional intelligence’: lessons from lesions. *Trends Neurosci.* 39 694–705. 10.1016/j.tins.2016.08.007 27647325 PMC5807001

[B26] Hornak J. Bramham J. Rolls E. T. Morris R. G. O’Doherty J. Bullock P. R. (2003). Changes in emotion after circumscribed surgical lesions of the orbitofrontal and cingulate cortices. *Brain* 126 1691–1712. 10.1093/brain/awg168 12805109

[B27] Hwang W. Y. Chen N. S. Dung J. J. Yang Y. L. (2007). Multiple representation skills and creativity effects on mathematical problem solving using a multimedia whiteboard system. *Educ. Technol. Soc.* 10 191–212.

[B28] Killgore W. D. S. Weber M. Schwab Z. J. DelDonno S. R. Kipman M. Weiner M. R. (2012). Gray matter correlates of Trait and Ability models of emotional intelligence. *Neuroreport* 23 551–555. 10.1097/WNR.0b013e32835446f7 22546702

[B29] Kim E. Zeppenfeld V. Cohen D. (2013). Sublimation, culture, and creativity. *J. Pers. Soc. Psychol.* 105 639–666. 10.1037/a0033487 23834638

[B30] Kong F. Zhao J. J. (2013). Affective mediators of the relationship between trait emotional intelligence and life satisfaction in young adults. *Pers. Individ. Dif.* 54 197–201. 10.1016/j.paid.2012.08.028

[B31] Koven N. S. Roth R. M. Garlinghouse M. A. Flashman L. A. Saykin A. J. (2011). Regional gray matter correlates of perceived emotional intelligence. *Soc. Cogn. Affect. Neurosci.* 6 582–590. 10.1093/scan/nsq084 20934987 PMC3190210

[B32] Kreifelts B. Ethofer T. Huberle E. Grodd W. Wildgruber D. (2010). Association of trait emotional intelligence and individual fMRI-activation patterns during the perception of social signals from voice and face. *Hum. Brain Mapp.* 31 979–991. 10.1002/hbm.20913 19937724 PMC6871025

[B33] Kringelbach M. L. (2005). The human orbitofrontal cortex: linking reward to hedonic experience. *Nat. Rev. Neurosci.* 6 691–702. 10.1038/nrn1747 16136173

[B34] Krueger F. Barbey A. K. McCabe K. Strenziok M. Zamboni G. Solomon J. (2009). The neural bases of key competencies of emotional intelligence. *Proc. Natl. Acad. Sci. U.S.A.* 106 22486–22491. 10.1073/pnas.0912568106 20080795 PMC2799712

[B35] Li W. F. Li X. T. Huang L. J. Kong X. Z. Yang W. J. Wei D. T. (2015). Brain structure links trait creativity to openness to experience. *Soc. Cogn. Affect. Neurosci.* 10 191–198. 10.1093/scan/nsu041 24603022 PMC4321617

[B36] Libbrecht N. Lievens F. Carette B. Cote S. (2014). Emotional intelligence predicts success in medical school. *Emotion* 14 64–73. 10.1037/a0034392 24219393

[B37] Lin C. Wang M. (1994). *The Creativity Assessment Packet.* Taipei: Psychological Publishing.

[B38] Mayer J. D. Roberts R. D. Barsade S. G. (2008). Human abilities: emotional intelligence. *Annu. Rev. Psychol.* 59 507–536. 10.1146/annurev.psych.59.103006.093646 17937602

[B39] Medford N. Critchley H. D. (2010). Conjoint activity of anterior insular and anterior cingulate cortex: awareness and response. *Brain Struct. Funct.* 214 535–549. 10.1007/s00429-010-0265-x 20512367 PMC2886906

[B40] Moon C. M. Jeong G. W. (2015). Alterations in white matter volume and its correlation with clinical characteristics in patients with generalized anxiety disorder. *Neuroradiology* 57 1127–1134. 10.1007/s00234-015-1572-y 26293129

[B41] Onur E. Alkin T. Sheridan M. J. Wise T. N. (2013). Alexithymia and emotional intelligence in patients with panic disorder, generalized anxiety disorder and major depressive disorder. *Psychiatr. Q.* 84 303–311. 10.1007/s11126-012-9246-y 23076764

[B42] Pan W. G. Wang T. Wang X. P. Hitchman G. Wang L. J. Chen A. T. (2014). Identifying the core components of emotional intelligence: evidence from amplitude of low-frequency fluctuations during resting state. *PLoS One* 9:e111435. 10.1371/journal.pone.0111435 25356830 PMC4214743

[B43] Parke M. R. Seo M. G. Sherf E. N. (2015). Regulating and facilitating: the role of emotional intelligence in maintaining and using positive affect for creativity. *J. Appl. Psychol.* 100 917–934. 10.1037/a0038452 25528247

[B44] Parker J. D. A. Summerfeldt L. J. Taylor R. N. Kloosterman P. H. Keefer K. V. (2013). Problem gambling, gaming and Internet use in adolescents: relationships with emotional intelligence in clinical and special needs samples. *Pers. Individ. Dif.* 55 288–293. 10.1016/j.paid.2013.02.025

[B45] Petrovic P. Ekman C. J. Klahr J. Tigerstrom L. Ryden G. Johansson A. G. M. (2016). Significant grey matter changes in a region of the orbitofrontal cortex in healthy participants predicts emotional dysregulation. *Soc. Cogn. Affect. Neurosci.* 11 1041–1049. 10.1093/scan/nsv072 26078386 PMC4927027

[B46] Piffer D. (2012). Can creativity be measured? An attempt to clarify the notion of creativity and general directions for future research. *Think. Skills Creat.* 7 258–264. 10.1016/j.tsc.2012.04.009

[B47] Preacher K. J. Hayes A. F. (2004). SPSS and SAS procedures for estimating indirect effects in simple mediation models. *Behav. Res. Methods Instr. Comput.* 36 717–731. 10.3758/BF03206553 15641418

[B48] Preacher K. J. Hayes A. F. (2008). Asymptotic and resampling strategies for assessing and comparing indirect effects in multiple mediator models. *Behav. Res. Methods* 40 879–891. 10.3758/BRM.40.3.879 18697684

[B49] Rivers S. E. Brackett M. A. Reyes M. R. Mayer J. D. Caruso D. R. Salovey P. (2012). Measuring emotional intelligence in early adolescence with the MSCEIT-YV: psychometric properties and relationship with academic performance and psychosocial functioning. *J. Psychoeduc. Assess.* 30 344–366. 10.1177/0734282912449443

[B50] Roberts A. C. Wallis J. D. (2000). Inhibitory control and affective processing in the prefrontal cortex: neuropsychological studies in the common marmoset. *Cereb. Cortex* 10 252–262. 10.1093/cercor/10.3.252 10731220

[B51] Roelofs K. Minelli A. Mars R. B. van Peer J. Toni I. (2009). On the neural control of social emotional behavior. *Soc. Cogn. Affect. Neurosci.* 4 50–58. 10.1093/scan/nsn036 19047074 PMC2656885

[B52] Rudebeck P. H. Saunders R. C. Prescott A. T. Chau L. S. Murray E. A. (2013). Prefrontal mechanisms of behavioral flexibility, emotion regulation and value updating. *Nat. Neurosci.* 16 1140–1145. 10.1038/nn.3440 23792944 PMC3733248

[B53] Sahin F. Ozer E. Deniz M. E. (2016). The predictive level of emotional intelligence for the domain-specific creativity: a study on gifted students. *Egitim Ve Bilim* 41 181–197.

[B54] Salguero J. M. Palomera R. Fernandez-Berrocal P. (2012). Perceived emotional intelligence as predictor of psychological adjustment in adolescents: a 1-year prospective study. *Eur. J. Psychol. Educ.* 27 21–34. 10.1007/s10212-011-0063-8

[B55] Salovey P. Mayer J. D. (1990). Emotional intelligence. *Imagin. Cogn. Pers.* 9 185–211. 10.2190/DUGG-P24E-52WK-6CDG

[B56] Saver J. L. Damasio A. R. (1991). Preserved access and processing of social knowledge in a patient with acquired sociopathy due to ventromedial frontal damage. *Neuropsychologia* 29 1241–1249. 10.1016/0028-3932(91)90037-9 1791934

[B57] Schutte N. S. Malouff J. M. Hall L. E. Haggerty D. J. Cooper J. T. Golden C. J. (1998). Development and validation of a measure of emotional intelligence. *Pers. Individ. Dif.* 25 167–177. 10.1016/s0191-8869(98)00001-4

[B58] Silvers J. A. Hubbard A. D. Chaudhury S. Biggs E. Shu J. Grunebaum M. F. (2016). Suicide attempters with Borderline Personality Disorder show differential orbitofrontal and parietal recruitment when reflecting on aversive memories. *J. Psychiatr. Res.* 81 71–78. 10.1016/j.jpsychires.2016.06.020 27392071 PMC5021587

[B59] Silvia P. J. Christensen A. P. Cotter K. N. (2016). “Commentary: the development of creativity-ability, motivation, and potential,” in *Perspectives on Creativity Development* Vol. 151 ed. Barbot B. (Hoboken, NJ: John Wiley & Sons), 111–119. 10.1002/cad.20147 26994729

[B60] Stalnaker T. A. Cooch N. K. Schoenbaum G. (2015). What the orbitofrontal cortex does not do. *Nat. Neurosci.* 18 620–627. 10.1038/nn.3982 25919962 PMC5541252

[B61] Sternberg R. J. (1999). *Handbook of Creativity.* New York, NY: Cambridge University Press.

[B62] Takeuchi H. Taki Y. Nouchi R. Sekiguchi A. Hashizume H. Sassa Y. (2013a). Resting state functional connectivity associated with trait emotional intelligence. *Neuroimage* 83 318–328. 10.1016/j.neuroimage.2013.06.044 23792978

[B63] Takeuchi H. Taki Y. Sassa Y. Hashizume H. Sekiguchi A. Fukushima A. (2011). Regional gray matter density associated with emotional intelligence: evidence from voxel-based morphometry. *Hum. Brain Mapp.* 32 1497–1510. 10.1002/hbm.21122 20740644 PMC6870144

[B64] Takeuchi H. Taki Y. Sassa Y. Hashizume H. Sekiguchi A. Nagase T. (2013b). White matter structures associated with emotional intelligence: evidence from diffusion tensor imaging. *Hum. Brain Mapp.* 34 1025–1034. 10.1002/hbm.21492 22139821 PMC6870451

[B65] Tan Y. F. Zhang Q. L. Li W. F. Wei D. T. Qiao L. Qiu J. (2014). The correlation between Emotional Intelligence and gray matter volume in university students. *Brain Cogn.* 91 100–107. 10.1016/j.bandc.2014.08.007 25282329

[B66] Toyama H. Mauno S. (2017). Associations of trait emotional intelligence with social support, work engagement, and creativity in Japanese eldercare nurses. *Jpn. Psychol. Res.* 59 14–25. 10.1111/jpr.12139

[B67] Vuilleumier P. Richardson M. P. Armony J. L. Driver J. Dolan R. J. (2004). Distant influences of amygdala lesion on visual cortical activation during emotional face processing. *Nat. Neurosci.* 7 1271–1278. 10.1038/nn1341 15494727

[B68] Wang C. K. (2002). The relationship between emotional intelligence and anxiety, depression and mood in a sample of college students. *Chin. J. Clin. Psychol.* 10 298–299.

[B69] Wang D. (2007). A report on the third revision of Combined Raven’s Test (CRT-C3) for children in China. *Chin. J. Clin. Psychol.* 15 559–568.

[B70] Williams F. E. (1980). *Creativity Assessment Packet (CAP): Manual.* Buffalo, NY: DOK.

[B71] Wolf R. C. Philippi C. L. Motzkin J. C. Baskaya M. K. Koenigs M. (2014). Ventromedial prefrontal cortex mediates visual attention during facial emotion recognition. *Brain* 137 1772–1780. 10.1093/brain/awu063 24691392 PMC4032099

[B72] Yao X. N. Yuan S. G. Yang W. J. Chen Q. L. Wei D. T. Hou Y. L. (2017). Emotional intelligence moderates the relationship between regional gray matter volume in the bilateral temporal pole and critical thinking disposition. *Brain Imaging Behav.* 12 488–498. 10.1007/s11682-017-9701-3 28357535

[B73] Zavala M. A. Lopez I. (2012). Adolescents at risks: what is the role of emotional intelligence? *Behav. Psychol.* 20 59–75.

[B74] Zeng L. Salvendy G. Zhang M. (2009). Factor structure of web site creativity. *Comput. Hum. Behav.* 25 568–577. 10.1016/j.chb.2008.12.023

[B75] Zhuang K. X. Xia Y. M. Sun J. Z. Chen Q. L. Wei D. T. Yang W. J. (2017). Emotion-related brain structures associated with trait creativity in middle children. *Neurosci. Lett.* 658 182–188. 10.1016/j.neulet.2017.08.008 28780167

